# Lung Cancer Screening: All That Glitters Is Not Gold

**DOI:** 10.7759/cureus.57783

**Published:** 2024-04-07

**Authors:** Sai Doppalapudi, Sindhaghatta Venkatram, Aam A Baqui, Gilda Diaz-Fuentes

**Affiliations:** 1 Pulmonary and Critical Care Medicine, BronxCare Hospital, Bronx, USA; 2 Pathology, BronxCare Hospital, Bronx, USA; 3 Pulmonary and Critical Care, BronxCare Hospital, Bronx, USA

**Keywords:** cancer screening, mycobacterium tuberculosis, pet/ct, granulomas, low-dose ct, lung cancer

## Abstract

Lung cancer screening with low-dose computed tomography (LDCT) can significantly improve survival rates with early detection. With the increased amount of imaging studies being performed for screening, there are more incidental lesions found. Malignancy and pulmonary infections are two of the major differentials when a lesion is found on CT. Neither a CT scan nor a positron emission tomography can reliably differentiate between malignancy and infectious lesions. Here, we present an unexpected case of multiple nodules detected on LDCT that was performed for lung cancer screening and the workup that was done to lead to a diagnosis.

## Introduction

Lung cancer is the number one cause of cancer-related deaths in the United States. The mortality for lung cancer is predictably much lower in those with early-stage compared to later-stage cancer. Lung cancer screening involves the use of low-dose computed tomography (LDCT) for the detection of lesions that represent malignancy. The goal is to identify cancer at an early stage when it is more easily treatable. One of the primary harms of any screening modality is false-positive tests, along with its resultant diagnostic workup and complications thereof. In the National Lung Screening Trial, the false positive rate using computed tomography was approximately 27%. Most incidental findings are benign and clinically insignificant. The most common findings are pulmonary (69%), cardiovascular (67%), and gastrointestinal (25%), but some require urgent recognition and further management [1].

## Case presentation

A 61-year-old Hispanic man, with a history of more than 30 pack-years of smoking and recreational drug use, was evaluated in the pulmonary clinic for a lung nodule found on LDCT. He was born and lived in New York City all his life, with occasional trips to Puerto Rico, the last one more than a year before presentation. The patient had no symptoms except for a weight loss of around 20 pounds in the past year. He denied exposure to sick contacts.

Physical examination findings

On initial evaluation, the patient appeared in good health and well-groomed, he was afebrile and had normal blood pressure, heart rate, and respiratory rate. His oxygen saturation was 99% on ambient air. No signs of temporal wasting, peripheral edema, or clubbing were observed. Chest auscultation was clear with equal bilateral breath sounds without any abnormal sounds; cardiac examination confirmed a normal and regular heartbeat without murmurs. The rest of the exam was normal. No cervical or axillary lymphadenopathy was found.

Diagnostic studies

**Figure 1:**
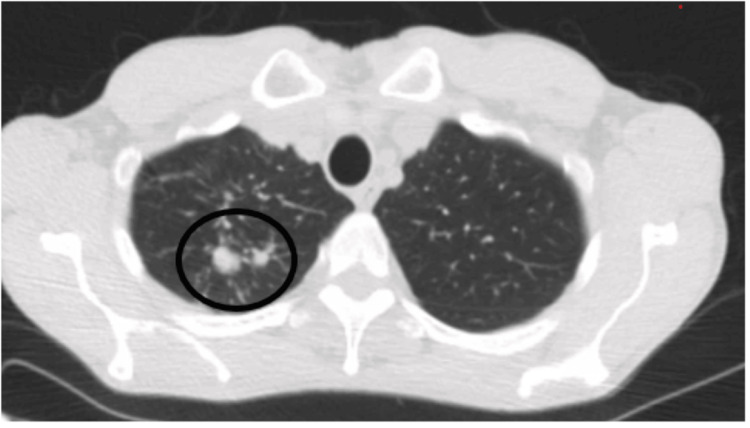
CT scan (axial): right upper 2 spiculated nodules measuring 24 mm and 17.8 mm

**Figure 2:**
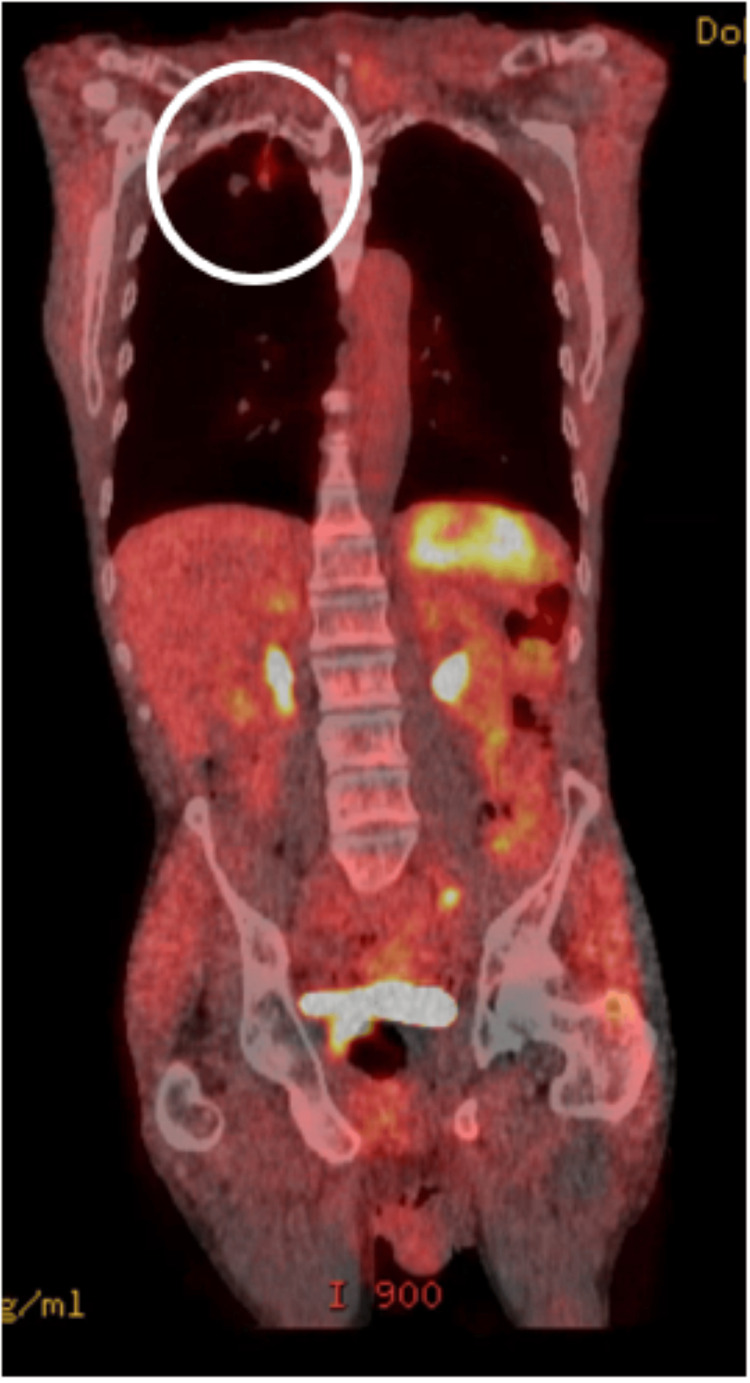
PET scan coronal view: right upper lobe nodule with SUVmax 4 PET: positron emission tomography; SUVmax: maximum standardized uptake value

**Figure 3:**
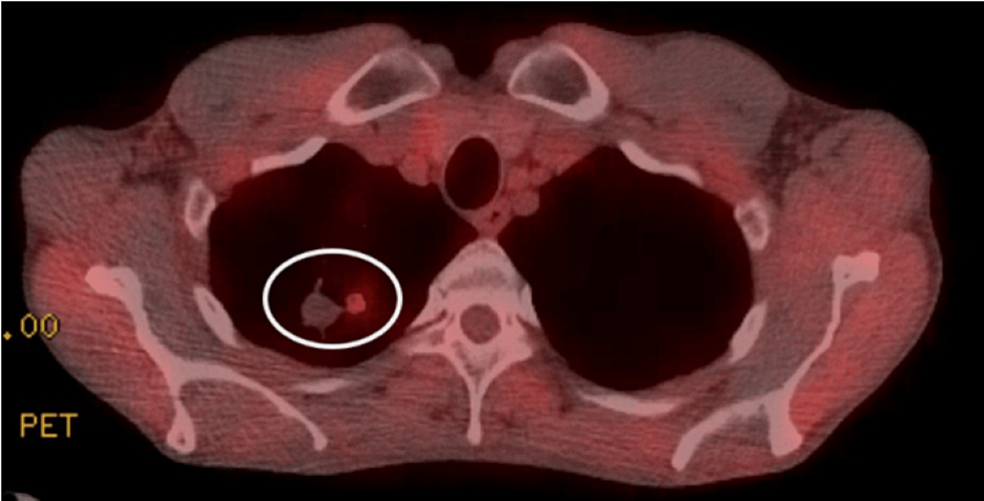
PET scan axial view: right upper lobe nodule with an SUVmax of 4 PET: positron emission tomography; SUVmax: maximum standardized uptake value

The LDCT showed multiple solid lung nodules with spiculated margins in the right upper lobe with the two largest ones measuring 24 mm and 17.8 mm along with centrilobular emphysema (Figure 1). There was no mediastinal or hilar lymphadenopathy identified. He had Lung-RADS® (Lung Imaging Reporting and Data System) category 4 and a 40.26% probability of malignancy calculated by the Brock University cancer prediction equation. There was no prior imaging to compare with. Given the high suspicion of malignancy, he underwent a positron emission tomography (PET) CT imaging, which showed metabolic activity with an SUVmax (maximum standardized uptake value) of 4 in the right upper lobe (Figures 2, 3). There were no fluorodeoxyglucose-avid axillary, mediastinal, or hilar lymph nodes. The revised probability of malignancy based on the Herder model was 96%.

**Figure 4:**
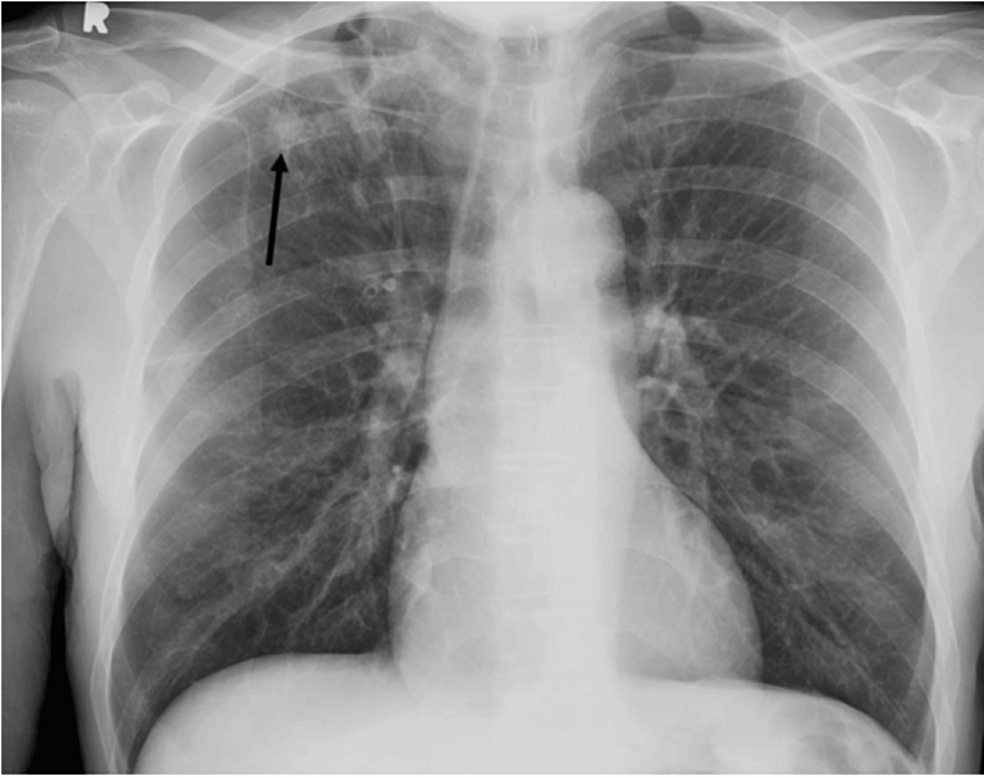
Chest X-ray: right upper lobe nodule

**Figure 5:**
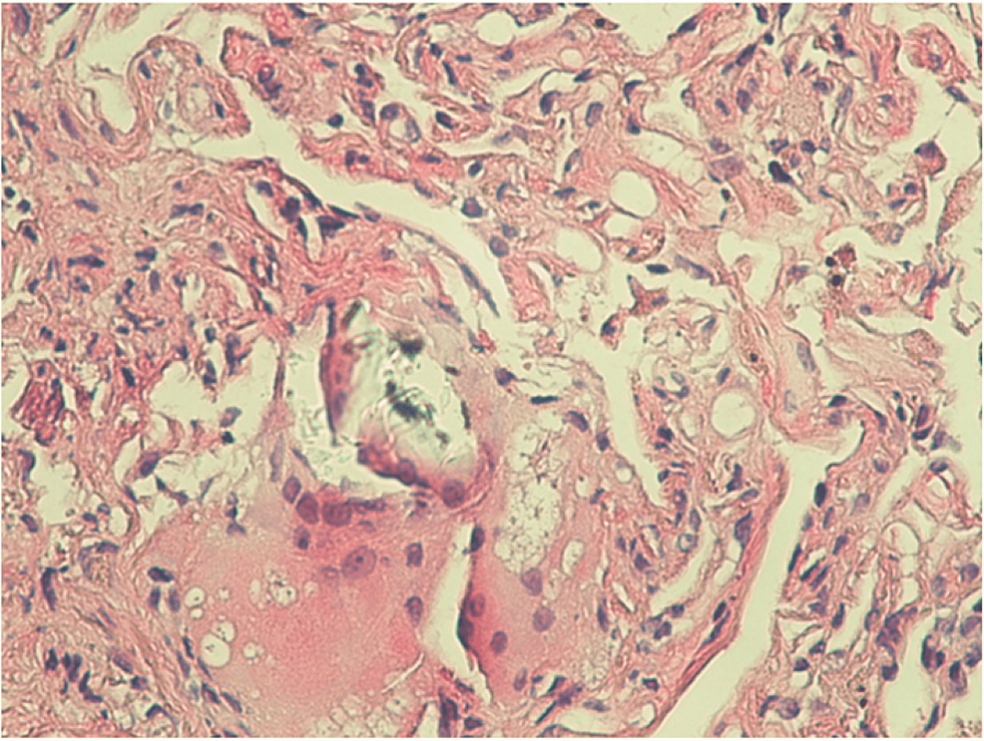
Pathology slide: bronchial mucosa with subepithelial chronic inflammation, fibroelastosis, and lung tissue with ill-formed granuloma and multinucleated giant cells

He underwent a fiberoptic bronchoscopy with endobronchial ultrasound, needle aspiration of hilar lymph nodes, and transbronchial biopsies of the nodules. Visual inspection of the tracheobronchial tree with bronchoscopy showed normal mucosa, minimal secretions, and no endobronchial lesions. Transbronchial biopsies of the pulmonary nodule and bronchoalveolar lavage from the right upper lobe were performed. Chest X-ray after bronchoscopy is shown in Figure 4. Pathology from transbronchial biopsies revealed non-necrotizing granuloma (Figure 5). Mycobacteria cultures were positive for Mycobacterium tuberculosis.

## Discussion

LDCT for lung cancer screening: good, bad, and surprising!

Lung cancer remains the leading cause of cancer-related mortality globally, with many cases diagnosed at an advanced stage, limiting treatment effectiveness. Early detection is crucial, and screening with LDCT offers a promising tool for the early identification of lung cancer, especially in high-risk populations [2].

The Good

The early detection of lung cancer through LDCT can significantly improve survival rates by identifying the disease when curative interventions are more likely to be successful. The United States Preventive and Screening Task Force recommends LDCT screening for adults aged 50 to 80 years who have a history of smoking greater than 20 pack years and currently smoke or have quit within the past 15 years [3]. This recommendation is based on substantial clinical evidence highlighting the efficacy of LDCT in detecting lung cancer early, particularly among high-risk individuals.

The Bad

Despite its benefits, LDCT screening poses significant challenges, notably the high rate of false positives. Non-cancerous nodules are often detected, leading to unnecessary anxiety, further invasive testing, and procedures with their own risks. The National Lung Screening Trial highlighted that approximately 96% of positive LDCT screenings were false positives, underscoring the need for improved specificity in lung cancer screening methods [2]. In the NLST, for every 1,000 individuals screened, false positives resulted in 17 invasive procedures (with 59 as the number needed to harm) and less than one major complication per person [4].

The Surprising

LDCT screenings sometimes reveal unexpected findings unrelated to lung cancer, known as "incidental findings." The reported prevalence ranges from 1% to 19% and as high as 59% in recent publications, explained by a lack of standards while reporting [5-7]. These can range from benign conditions to serious, potentially life-threatening diseases. The incidence of infection is reported in 6% of patients and radiological findings include ground glass opacifications, consolidations, and tree-in-bud opacities [8].

The granuloma paradox

A granuloma is a compact aggregate of histiocytes [9]. Granulomas can be necrotizing or non-necrotizing. Differential diagnoses of granulomas include infections like mycobacterial, fungal, and parasitic organisms and noninfectious causes like sarcoidosis, berylliosis, hypersensitivity pneumonitis, and lymphocytic interstitial pneumonia among others. Malignancies associated with non-necrotizing granulomas have been reported in carcinomatosis and Hodgkin’s and non-Hopkins lymphomas. Granulomas associated with malignancy can be a secondary immune response to the tumor, a reaction to necrotic tumor cells or an unrelated cause coincidentally discovered during cancer evaluation. Special staining techniques are indispensable tools in the diagnosis and study of granulomas. They allow for the identification of infectious agents, differentiation of granuloma types, and understanding of the underlying pathology. The choice of stain depends on the suspected cause of the granuloma and the tissue type being examined. In our patient, the presence of non-necrotizing granuloma and negative special stains could not lead us to a specific differential [10].

PET positivity and tuberculosis

In our patient, PET positivity led to a scoring of high probability for lung cancer; PET scans are usually used to detect metabolic activity in lesions. A high metabolic activity generally indicates malignancy; however, inflammatory diseases can also result in positive metabolic activity. PET/CT imaging has shown considerable promise in assessing the extent of Mycobacterium tuberculosis infection and response to treatment [11]. Goo et al.'s research highlights the significant role of FDG PET scans in the diagnosis and management of pulmonary tuberculosis and tuberculomas, demonstrating that these lesions typically show increased FDG uptake (SUVmax value of 4.2 ± 2.2) [12]. In a tuberculosis endemic area, the diagnostic accuracy of PET for the differentiation of benign from malignant solitary pulmonary nodules was limited, SUV values were not significantly different in benign and malignant lesions [13]. SUV measurements from both tuberculous and malignant lesions tend to be high with significant overlap. SUV measurements are therefore not useful in characterizing lesions as granulomatous or malignant.

Tuberculosis in patients suspected to have lung cancer

Rolston et al. conducted a study on 2908 patients suspected of lung cancer; 93% of them had malignant tumors, 0.4% benign tumors, 1.3% infections (46% fungal, 27% mycobacterial, 22% bacterial, 5% parasitic), and the rest nonspecific findings [14]. Pitlik et al. found 26 cases of misdiagnosed neoplasms as tuberculosis in over 70,000 patients from 1973 to 1982, with no cancer detected upon invasive testing [14].

The paradox of tuberculosis and lung cancer

Research shows a strong link between tuberculosis and lung cancer; tuberculosis may increase the risk for lung cancer due to its chronic inflammation and damage to lung tissues. This damage, characterized by granuloma formation and repeated inflammatory injuries, can trigger abnormal cell growth, leading to cancer. Tuberculosis fosters an inflammatory environment conducive to tumor growth, with cytokines like interferon-gamma (IFN-γ), interleukin (IL)-1, IL-2, IL-12, and TNF playing a role, suggesting that Mycobacterium tuberculosis might encourage cancer through these inflammatory pathways [15]. Tuberculosis and lung cancer can coexist. A retrospective study of patients with both active tuberculosis and lung cancer from January 2016 to August 2021 revealed that survival rates varied according to the diagnosis interval. Patients diagnosed with lung cancer within six months showed different characteristics and outcomes compared to those diagnosed after six months, with the latter group generally exhibiting better survival rates. The study highlights the significance of early diagnosis and how the interval between Tuberculosis and cancer diagnoses can affect patient outcomes​ [16].

Back to our case

Our patient was diagnosed with pulmonary tuberculosis, and he is responding well to the standard four-drug regimen (rifampin, isoniazid, pyrazinamide, and ethambutol). He will be followed in pulmonary and infectious disease clinics with serial imaging and mycobacterium cultures.

## Conclusions

Our case highlights a surprising diagnosis: pulmonary tuberculosis in a patient initially screened for lung cancer due to a high-risk profile.

LDCT screening plays a pivotal role in the early detection of lung cancer, offering a significant potential to improve survival rates. However, the challenges of false positives and the management of incidental findings require careful consideration and balance. The discovery of incidental findings raises important considerations for clinical management within lung cancer screening programs. While these findings can complicate the screening process, they also offer opportunities for early intervention in conditions that may otherwise go undetected.
